# Silexan for treatment of psychiatric manifestations in the context of COVID-19: a case series

**DOI:** 10.1192/j.eurpsy.2023.1690

**Published:** 2023-07-19

**Authors:** L. Bartova, M. Dold, G. Fugger, A. Weidenauer, D. Rujescu-Balcu, S. Kasper

**Affiliations:** Department of Psychiatry and Psychotherapy, Medical University of Vienna, Wien, Austria

## Abstract

**Introduction:**

Silexan manufactured from *Lavandula angustifolia* showed favorable safety and efficacy in subthreshold and generalized anxiety disorder (GAD), mixed anxiety and depressive disorder (MADD), and further, especially subsyndromal psychiatric manifestations including depression, sleep disturbances, restlessness, fatigue and pain.

**Objectives:**

Since the abovementioned clinical phenotypes were repeatedly observed in the course of the so-called post coronavirus-19 disease (COVID-19) syndrome, which were, importantly, of subsyndromal severity in the most cases, we were confident that Silexan will be efficacious also in this indication.

**Methods:**

We report on three adult outpatients treated with Silexan due to psychiatric conditions that occurred in the context of a mild and short-lasting COVID-19 infection.

**Results:**

A 38-years old female experienced fatigue, brain fog, inner tension, restlessness and sad mood with weepiness after recovery of her respiratory COVID-19 infection. Since she did not remit under ongoing psychotherapy (PT), Silexan 80 mg p.o.q.d. was additionally employed and very well tolerated. She achieved full remission of her subthreshold symptoms within one month. A 27-years old male developed GAD including anxiety, inner tension, restlessness, irritability, muscle aches, difficulties in concentrating and in controlling feelings of worry after he recovered from respiratory COVID-19. Following his preference, PT and Silexan 80 mg p.o.q.d. were initiated and very well tolerated. Because of partial response, Silexan was increased to 80 mg twice daily after three weeks. After additional two weeks, he was able to enjoy everyday activities and to comply with working demands without relevant difficulties. A 38-years old female developed a post COVID-19 syndrome with fatigue, anxiety, depression, inner tension, tachycardia, hopelessness and rumination. Due to the current MADD Bupropion 150 mg and subsequently 300 mg p.o.q.d. and Hydroxyzine 25 mg on demand were administered. Although clinical improvement was achieved, the patient discontinued the treatment due to subjective exacerbation of tachycardia and refused any further treatment optimization. Once she agreed to phyto-psychopharmacotherapy, Silexan 80 mg p.o.q.d. was employed and, while well tolerated, increased to 80 mg twice daily after two weeks. The patient was increasingly able to participate at her working and social activities again and stayed stable for four months.

**Conclusions:**

To our knowledge, this is the first report on administering Silexan in subsyndromal and full-blown anxiety and depression with cognitive and psychosomatic symptoms that occurred in the context of COVID-19. While substantial clinical improvements were achieved, no relevant adverse effects occurred.

**Disclosure of Interest:**

None Declared

